# Effectiveness and safety of probiotics on patients with severe acute pancreatitis: A systematic review and meta-analysis

**DOI:** 10.1097/MD.0000000000036454

**Published:** 2023-12-15

**Authors:** Zhiling Gao, Shuomiao Yin, Kui Jin, Weiqun Nie, Longmei Wang, Ling Cheng

**Affiliations:** a Department of Intensive Care Unit, The First Affiliated Hospital of Anhui University of Chinese Medicine, Hefei, Anhui Province, China.

**Keywords:** early enteral nutrition, meta-analysis, probiotics, severe acute pancreatitis

## Abstract

**Background:**

This meta-analysis aimed to assess the efficacy and safety of probiotics in conjunction with early enteral nutrition for the treatment of severe acute pancreatitis (SAP). This study focused on multiple clinical endpoints, including mortality rate, risk of organ failure, and duration of hospital stay.

**Methods:**

In accordance with the Preferred Reporting Items for Systematic Reviews and Meta-Analyses guidelines. The study adhered to the Patient, Intervention, Comparison, Outcome framework and utilized randomized controlled trials to examine the impact of probiotics on patients with SAP. Data extraction and quality assessment were conducted independently by 2 evaluators, with discrepancies resolved collaboratively, or by a third adjudicator. Statistical analyses were performed using chi-square statistics, *I*^2^ metrics, and both fixed- and random-effects models, as dictated by heterogeneity levels.

**Results:**

The meta-analysis covered 6 randomized controlled trials. Compared to control groups (placebo or standard care without probiotics), probiotics did not significantly reduce mortality rates or organ failure risk. However, they notably shortened hospital stays by a weighted mean difference of −5.49 days (95% confidence interval: −10.40 to −0.58; *P* = .010). The overall bias risk was low to moderate.

**Conclusions:**

Probiotics combined with early enteral nutrition did not significantly improve mortality rates or reduce the risk of organ failure in patients with SAP, but shortened hospital stays. Further studies are required to corroborate these findings.

## 1. Introduction

Severe acute pancreatitis (SAP) is one of the most life-threatening emergencies encountered in the domain of gastrointestinal disease. Characterized by the rapid onset of inflammation in the pancreatic tissue and surrounding organs, this condition is precipitated by various factors that culminate in premature activation of pancreatic enzymes within the pancreatic acini. The course of SAP is often aggressive and marked by high morbidity and mortality rates, posing a grave risk to the affected individual’s life.^[1,2]^ Emerging research has pointed toward several pivotal pathophysiological mechanisms that contribute to SAP severity. A burgeoning body of evidence has indicated that increased intestinal permeability and subsequent impairment of the intestinal mucosal barrier are instrumental in the pathogenesis of SAP.^[3,4]^ Such disruptions lead to a phenomenon known as bacterial translocation from the intestinal lumen to the systemic circulation, further exacerbating the condition by inducing endotoxemia. These physiological disturbances act as catalysts that significantly increase morbidity and mortality associated with SAP.

Current clinical guidelines advocate the use of early enteral nutrition for the management of SAP. Early enteral nutrition has been found to preserve the integrity of the intestinal mucosal barrier and facilitate functional recovery.^[5,6]^ Thus, it serves as a preventive measure against bacterial translocations. However, the role of early enteral nutrition in selectively modulating the intestinal microbiota, particularly in eliminating pathogenic bacteria, remains poorly understood. Probiotics, as microbiota-modulating agents, have been the subject of increasing interest in the medical community because of their potential role in regulating the intestinal flora. These micro-organisms are thought to enrich the population of beneficial bacteria within the gastrointestinal tract, thereby inhibiting the proliferation and invasion of pathogenic species.^[7]^ Several studies have initiated the combined use of probiotics and early enteral nutrition in treating SAP; nevertheless, the efficacy and safety of this therapeutic strategy remain a matter of considerable debate.^[8]^

Thus, the present systematic review and meta-analysis aimed to critically examine the existing literature on the effectiveness and safety of employing probiotics in the treatment regimen for patients with severe acute pancreatitis. We intend to provide a comprehensive understanding of the clinical implications of such an approach, with a focus on patient outcomes and integrity of the gastrointestinal mucosal barrier.

## 2. Materials and methods

Before delving into the materials and methods, it is imperative to articulate the research objectives that drove this systematic review. The primary objective was to discern the effectiveness and safety of probiotics in the treatment of SAP. Stemming from this overarching objective, the following research questions were formulated: Does the adjunctive use of probiotics in SAP treatment significantly reduce patient mortality rates when compared to standard care without probiotics? Is the risk of organ failure in SAP patients altered (either increased or reduced) with the inclusion of probiotics in their treatment regimen? Does the integration of probiotics in the treatment protocol for SAP patients influence the duration of their hospital stay? Are there any significant adverse effects or safety concerns associated with the use of probiotics in SAP patients? These research questions informed our search strategy, inclusion/exclusion criteria, and the subsequent data extraction and analysis processes.

### 2.1. Search strategy

In conducting this systematic review and subsequent data synthesis, we rigorously complied with the guidelines set forth by the Preferred Reporting Items for Systematic Reviews and Meta-Analyses.^[9]^

To ensure a thorough and unbiased retrieval of pertinent studies, we executed a multi-database search on July 19, 2023, without temporal restrictions. The databases included PubMed, Embase, Web of Science, and the Cochrane Library. The search strategy employed a carefully selected array of key terms, namely severe acute pancreatitis, probiotics, and enteral nutrition, to align coherently with the expansive scope of the Patient, Intervention, Comparison, Outcome (PICO) framework. No limitations were imposed based on language. Additionally, the bibliographies of the relevant articles were manually scrutinized to identify any further records that could contribute to this meta-analysis.

### 2.2. Inclusion criteria and exclusion criteria

The meta-analysis was designed and structured based on the Patient, Intervention, Comparison, Outcome framework to meticulously address the following variables: Patient Population (P) consists of individuals clinically diagnosed with SAP; Intervention (I) entails the utilization of probiotics; comparison (C) incorporates either the conventional standard of care without probiotic supplementation or placebo treatment; and outcome (O) focuses on the efficacy of mitigating the clinical manifestations and complications associated with SAP, in addition to the safety profile of probiotic intervention.

The inclusion criteria were as follows: (1) Published randomized controlled trials focused on the clinical effects of probiotics combined with early enteral nutrition for the treatment of SAP; and (2) study subjects must have a confirmed diagnosis of SAP. Baseline characteristics between the intervention and control groups must be consistent to ensure comparability. (3) Intervention and Control Measures: Control Group: The treatment modalities should include gastrointestinal decompression, maintenance of electrolyte balance, and parenteral nutrition. Intervention Group: The intervention group’s treatment should be supplemented with the addition of probiotics combined with early enteral nutrition, where “early” is defined as 24 to 72 hours after diagnosis.^[10]^ (4) Outcome Measures: The primary outcomes should be focused on mortality rate, risk of organ failure, and duration of hospital stay. Secondary outcomes can include inflammatory markers, quality of life scores, and any other pertinent clinical endpoints.

The exclusion criteria were as follows: (1) Studies where SAP diagnosis was not confirmed or in which baseline characteristics between intervention and control groups were not comparable; (2) studies with incomplete or ambiguous statistical data; and (3) nonempirical studies and descriptive literature: case reports, editorial commentaries, expert opinions, and narrative reviews were systematically excluded; (4) studies that did not report or measure the defined primary or secondary outcomes.

### 2.3. Study selection

Upon completing the initial database search, we screened the titles and abstracts of retrieved articles to exclude irrelevant studies. We then assessed the full-text articles of potentially relevant studies against the predefined inclusion and exclusion criteria. Any duplicates were removed to avoid redundancy. We meticulously documented the entire process of study selection, clearly annotating reasons for exclusions for full-text articles. A flow diagram, in accordance with Preferred Reporting Items for Systematic Reviews and Meta-Analyses guidelines, was constructed to visualize the selection process and to provide a clear breakdown of included and excluded studies.

### 2.4. Data extraction

In compliance with the stringent methodological criteria essential for medical meta-analyses, literature assessment and data extraction were performed independently by 2 evaluators and subsequently cross-validated to ensure fidelity and coherence. In cases of incongruities, the involved reviewers resolved discrepancies through collaborative dialogue; if the resolution proved elusive, a third adjudicator was consulted. Data parameters to be abstracted include study identification and publication year, detailed specifications of interventional modalities, the number of subjects randomized into treatment and control groups, as well as key demographic and clinical baseline attributes such as mean age (with standard deviation), sex distribution, and predictive indices including Acute Physiology and Chronic Health Evaluation II, Ranson criteria, and C-reactive protein levels. If the requisite data were missing from published articles, direct correspondence was initiated with the primary investigators via email to solicit unpublished or ancillary data. This consolidated passage adheres to academic standards specific to meta-analyses, while maintaining terminological accuracy and thoroughness.

### 2.5. Quality assessment

In accordance with rigorous methodological standards for medical meta-analyses, the quality of the included studies was evaluated using the Cochrane Collaboration’s risk of bias instrument.^[11]^ Two independent reviewers appraised the studies across 6 specific domains: random sequence generation, allocation concealment, blinding of participants and personnel, handling of incomplete outcome data, selective outcome reporting, and identification of other potential sources of bias. Each of these domains was categorized as presenting either a low, unclear, or high risk of bias. In instances where interpretive discordance occurred between the 2 reviewers, an attempt was made to reach a consensus through deliberative discussion; consultation with a third reviewer was sought if a resolution could not be achieved.

### 2.6. Statistical analyses

In adherence to stringent methodological protocols appropriate for meta-analytical research in medicine, statistical heterogeneity among the included studies was evaluated using chi-square statistics and expressed quantitatively using the *I*^2^ metric. An *I*^2^ value falling below 50% in conjunction with a *P* value equal to or exceeding .10 was interpreted as an absence of noteworthy heterogeneity, warranting the application of a fixed-effects model for the calculation of the aggregate effect size. Conversely, an *I*^2^ value of 50% or greater, or a corresponding *P* value below .10, was indicative of significant statistical heterogeneity, thereby necessitating the use of a random-effects model for the combined effect size computation. To scrutinize potential publication bias, the symmetry of the funnel plot was assessed. A symmetrical distribution of data points around the funnel plot’s apex would imply a lower probability of the meta-analysis results being skewed by publication bias. Furthermore, Egger linear regression test served as a quantitative method for detecting any existing publication bias. All statistical tests were 2-tailed, and a *P* value of <.05 was considered to be statistically significant. Statistical analyses were performed using Stata version 17 (StataCorp, College Station, TX).

## 3. Results

### 3.1. Search results and study selection

**Figure 1.**
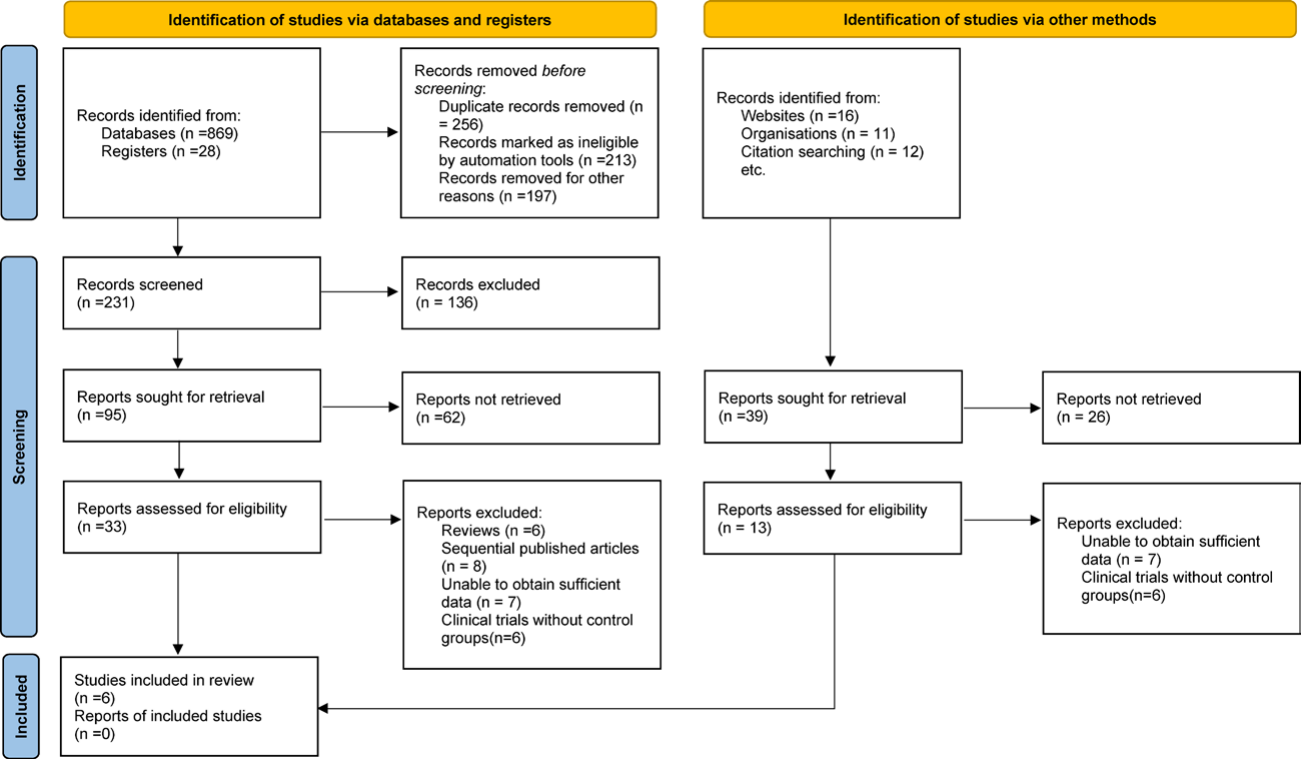
Selection process of included studies.

A total of 936 relevant articles were identified during the initial phase of our database query. Following the elimination of duplicate records and extensive review of titles and abstracts in alignment with our predefined inclusion and exclusion criteria, we narrowed the selection to 33 pertinent articles. Subsequent to a more exhaustive review, 27 of these were dismissed, resulting in a final count of 6 articles that were deemed eligible for inclusion in the meta-analysis.^[12–17]^ A detailed illustration of the literature screening workflow and its outcomes are shown in Figure 1.

### 3.2. Study characteristics

In the meta-analysis, 6 studies spanning from 2007 to 2017 were evaluated, involving a varied number of randomized participants, ranging from as few as 15/14 to as many as 62/61 in the treatment and control groups. These interventions predominantly explored the efficacy of bioactive fibers and various strains of probiotics administered in diverse formulations and frequencies. The mean age of the participants fluctuated across studies, ranging from 42.6 to 60.4 years. The male participation rate varied considerably, ranging from 28% to 81.8%. The Acute Physiology and Chronic Health Evaluation II scores were consistently moderate, while the Ranson criteria and C-reactive protein levels varied, although several studies did not provide these data. Overall, the included studies exhibited heterogeneity in the participant characteristics, interventions, and reporting of clinical metrics (Table 1).

**Table 1:** Characteristics of studies included in the meta-analysis.

Author	Year	No. of participants (randomized)	Intervention & control groups	Mean age (SD) (yr)	% Male	APACHE II score	Ranson criteria	CRP level (mg/L)
Wu et al^[17]^	2017	60/60	Treatment: Bifidobacterium quadruple living bacterium including 0.5 × 10^8^ *B. bifidus*, 0.5 × 10^8^ *B. acidophilus*, 0.5 × 10^8^ *E. faecalis*, 0.5 × 10^8^ *B. cereus*, thrice daily. Control: Standard enteral nutrition	42.7 (11.5)	56.7	11	5.1/4.9	NA
Wang et al^[16]^	2013	62/61	Treatment: 1.5 × 107 *B. subtilis* and 1.35 × 10^8^ *E. faecium* enteric-coated capsules, thrice daily. Control: Standard enteral nutrition	42.6 (13.8)	51.6	12	5.1/5.0	89/13.3
Plaudis et al^[15]^	2012	30/28	Treatment: Bioactive fibers (Synbiotic 2000 Forte twice daily). Control: Bioactive fibers	NA	28	8.8/8.6	NA	NA
Besselink et al^[12]^	2008	15/14	Treatment: 10^10^ *L. acidophilus, L. casei, L. salivarius, L. lactis, B. bifidum*, and *B. lactis* in a total daily dose (Ecologic 641), twice daily for 28 d. Control: Placebo	60.4 (16.5)	59.9	8.6/8.4	NA	268/270
Olah et al^[14]^	2007	33/47	Treatment: Bioactive fibers (Synbiotic 2000 Forte once daily for 7 days). Control: Bioactive fibers	47.5 (19–78)	81.8	NA	N/A	216.7/191.2
Karakan et al^[13]^	2007	15/30	Treatment: 0.7 g/100 mL soluble fiber and 0.8 g/100 mL insoluble fiber. Control: Standard enteral nutrition	47.3 (16.8)	40	9.4/9.6	NA	232/244

APACHE II = Acute Physiology and Chronic Health Evaluation II score, CRP = C-reactive protein, NA = not available.

### 3.3. Results of quality assessment

**Figure 2.**
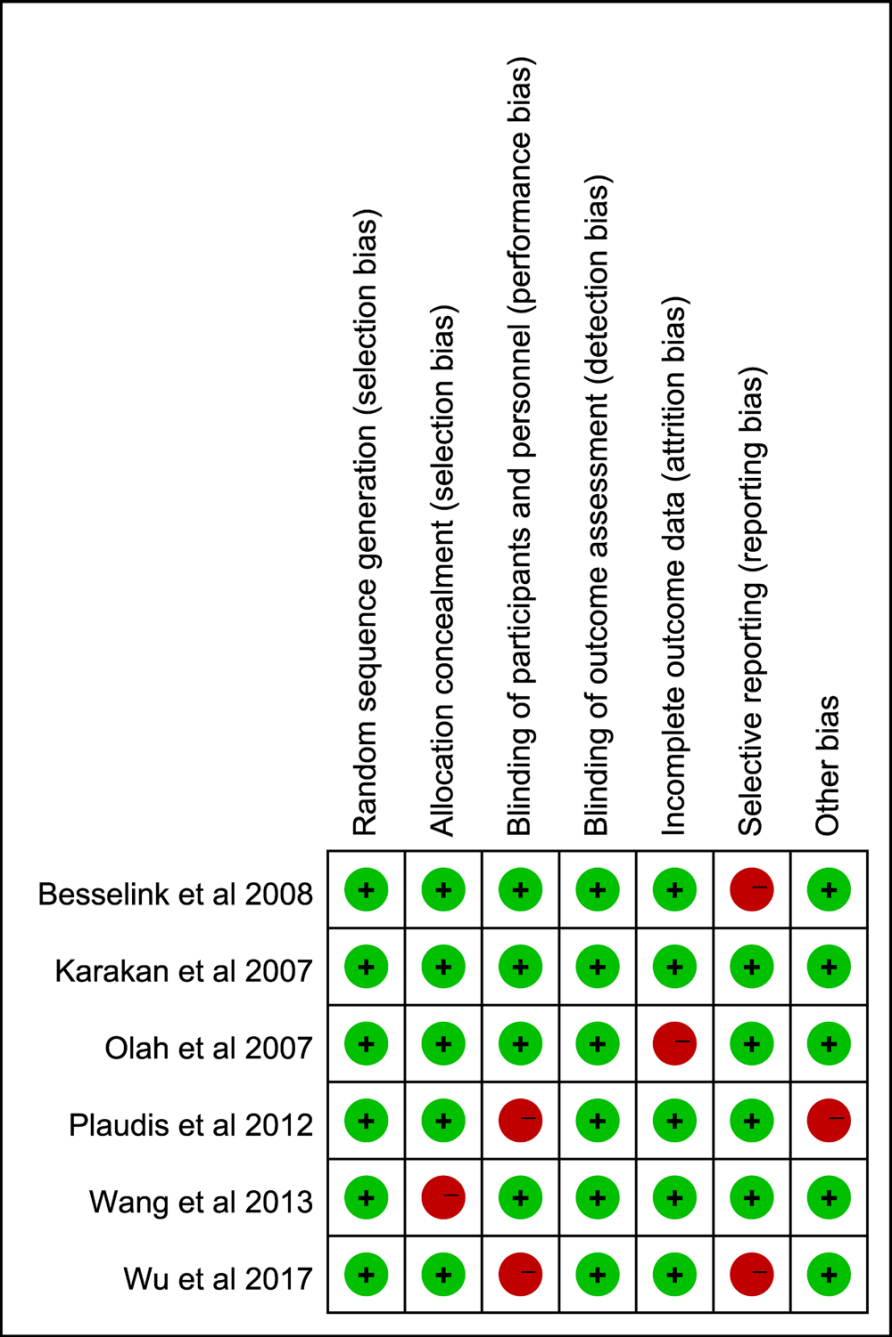
Quality assessment of included studies using Cochrane Collaboration’s tool criteria. Red in figure indicates high risk and green means low risk.

In the meta-analysis, the risk of bias was rigorously appraised across multiple dimensions within the 6 incorporated studies. One study demonstrated methodological robustness, exhibiting a low susceptibility to bias across all evaluated domains. Nonetheless, one-third of the studies (33.3%) manifested a heightened risk of bias concerning blinding of participants and personnel, thus raising concerns over potential performance bias that may have skewed the results. Additionally, an equivalent percentage (33.3%) of the included randomized controlled trials demonstrated an elevated risk of selective reporting bias. Such selective reporting could have introduced distortions into the cumulative findings of these studies (Fig. 2). The subsequent details encapsulate the findings: Random sequence generation (selection bias): All 6 studies satisfied this criterion, suggesting a robust randomization process. Allocation concealment (selection bias): 5 studies met this criterion, while one study presented potential risks, indicating possible knowledge of the forthcoming intervention in some studies. Blinding of participants and personnel (performance bias): 4 studies adequately blinded participants and personnel. However, 2 studies did not satisfy this criterion, implying potential performance bias. Blinding of outcome assessment (detection bias): All 6 studies met this criterion, signifying a reduced risk of detection bias in the outcomes reported. Incomplete outcome data (attrition bias): 5 studies exhibited comprehensive outcome data. In contrast, one study did not meet this criterion, raising concerns about the potential exclusion or loss of participants during the study. Selective reporting (reporting bias): 4 studies reported their findings comprehensively, whereas 2 studies were found to potentially omit or selectively report outcomes, which could introduce bias in the representation of results. Other bias: 5 studies exhibited no other discernible biases. However, one study had potential undisclosed biases that could influence the findings.

### 3.4. Impact of probiotics therapy on modality

**Figure 3.**
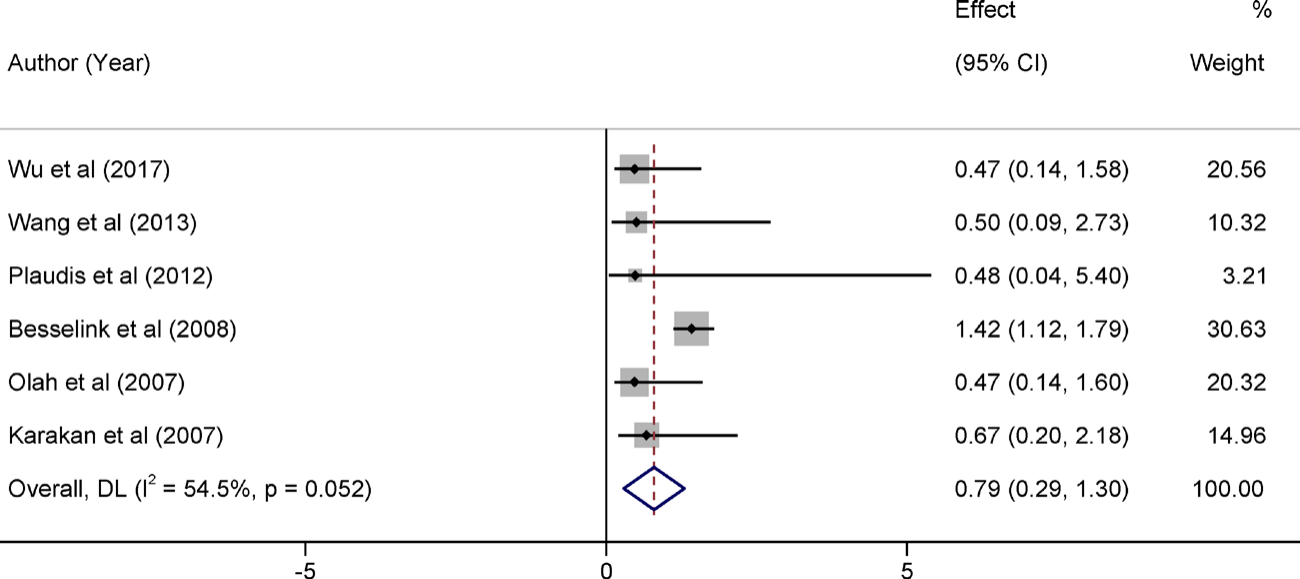
Forest plots of the impact of probiotics therapy on modality.

In a meta-analysis, the use of probiotics as a treatment modality did not result in a statistically significant reduction in mortality rates. The pooled relative risk was calculated as 0.79, falling within the 95% confidence interval (CI) of 0.29–1.30. Furthermore, the *I*^2^ statistic, which quantifies the heterogeneity among the included studies, was 54.5% (*P* = .052). This indicated a moderate level of heterogeneity in the outcomes across different trials. It is imperative to note that despite the absence of statistical significance, there was a trend toward reduced mortality in the intervention group, which could warrant further investigation (Fig. 3).

### 3.5. Impact of probiotics therapy on organ failure risk

**Figure 4.**
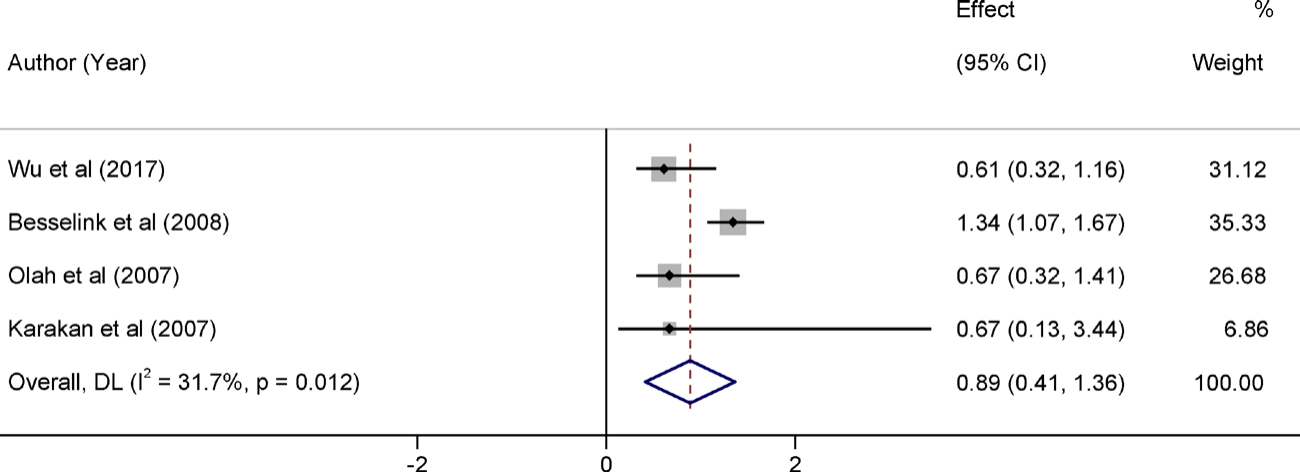
Forest plots of the impact of probiotics therapy on organ failure risk.

It is worth highlighting that the risk of organ failure is a vital clinical endpoint given its direct implications for patient morbidity and healthcare resource utilization. The *I*^2^ statistic, a quantitative measure of inter-study variability, was 31.7% (*P* = .012). This represents low to moderate heterogeneity among the included trials. When synthesizing the available data, the applied interventions failed to demonstrate a statistically significant effect on the reduction of organ failure. Specifically, the aggregated relative risk was determined to be 0.89, encapsulated by a 95% CI ranging from 0.41 to 1.36 (Fig. 4).

### 3.6. Efficacy of probiotics in reducing hospital stay duration

**Figure 5.**
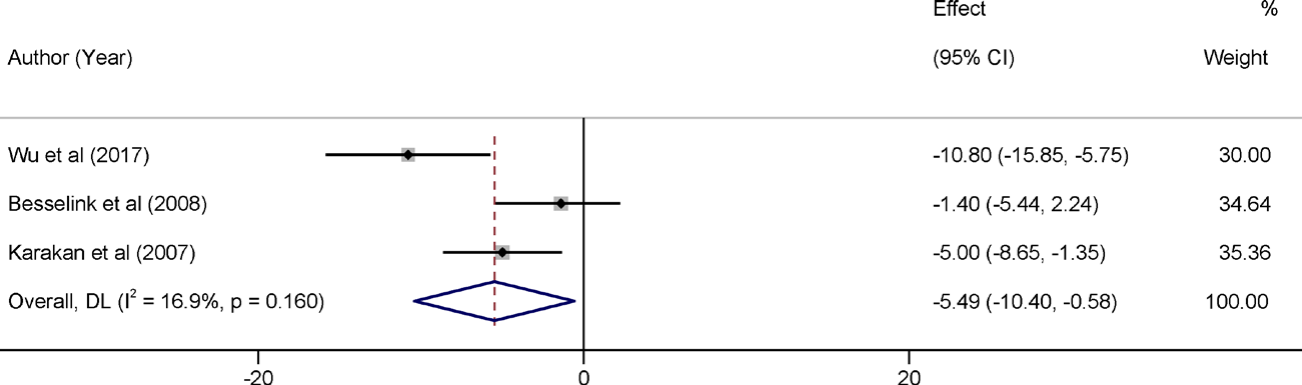
Forest plots of the impact of probiotics therapy on reducing hospital stay duration.

Upon systematic data aggregation and analysis, our findings indicate a statistically significant reduction in the duration of hospital stay for patients with SAP treated with probiotics. The weighted mean difference for the length of stay was found to be −5.49 days, with a 95% CI ranging from −10.40 to −0.58 days. It is vital to note that this observed effect was statistically significant, as evidenced by the *P* value of .010. Regarding study heterogeneity, an *I*^2^ statistic of 16.9% was observed, accompanied by a *P* value of .16 (Fig. 5). This relatively low *I*^2^ percentage indicated that the included studies were largely consistent in their findings, thereby bolstering the reliability of our pooled estimates.

### 3.7. Sensitivity analysis

**Figure 6.**
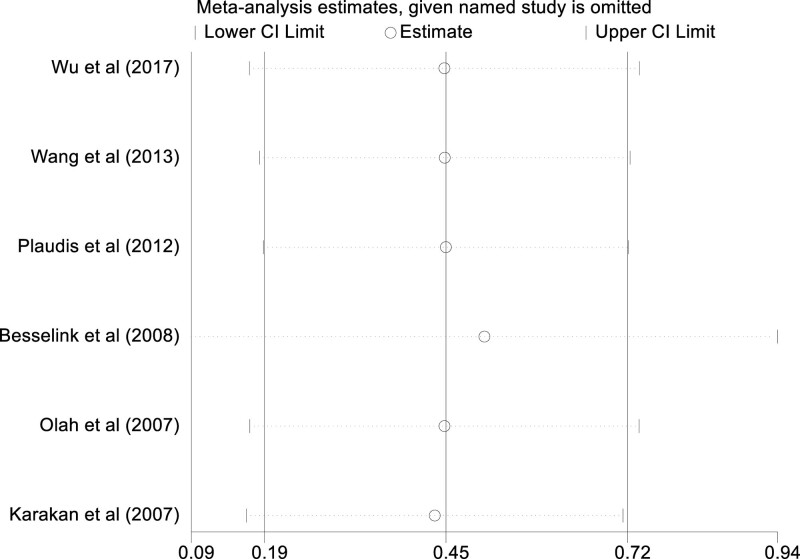
Sensitivity analysis of the impact of probiotics therapy on modality.

In light of the discernible heterogeneity observed across the studies included in this meta-analysis, we performed a methodical sensitivity analysis to evaluate the resilience and trustworthiness of the aggregated results. Employing a leave-one-out strategy, each study was sequentially omitted, followed by recalculation of the aggregated effect size utilizing the remaining studies. The outcomes of this stringent sensitivity analysis affirmed that the consolidated results were neither perturbed nor compromised by the exclusion of any individual study. This indicated that the influence of each study on the pooled results was proportionate, thereby amplifying the credence attributed to our aggregated findings. The enduring stability across these assessments corroborates the rigor of our primary outcomes, augmenting the validity of the conclusions deduced from this meta-analysis (Fig. 6).

### 3.8. Publication bias

**Figure 7.**
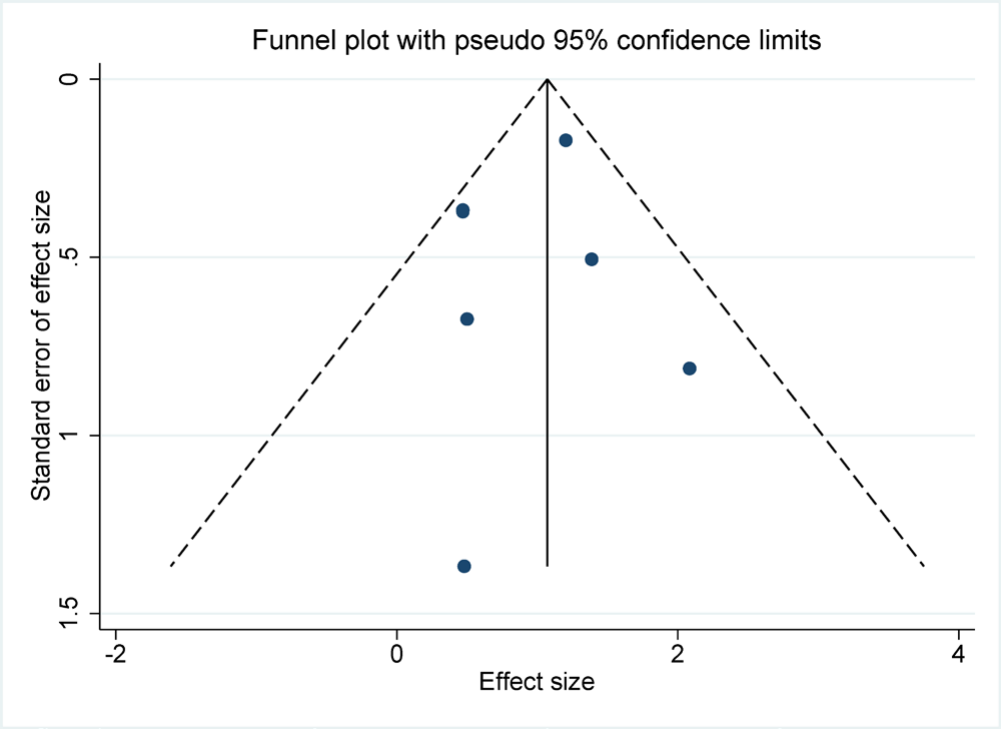
Funnel plot for publication bias in all included studies.

Given that our meta-analysis included only 6 studies, the funnel plot primarily serves as a visual tool for preliminary insights regarding potential publication bias. The funnel plots displayed a symmetrical distribution, suggesting an absence of notable publication bias (Fig. 7). Importantly, to bolster our evaluation, we conducted Egger linear regression test, a quantitative method that helps ascertain the potential asymmetry of the funnel plot. The outcomes from the Egger test did not indicate significant publication bias across the studied parameters (*P* values > .05 for all variables examined). Combined, these results uphold the credibility of the meta-analysis and emphasize the rigor of our synthesized findings.

## 4. Discussion

SAP is a debilitating inflammatory disorder initiated by aberrant activation of pancreatic enzymes, leading to rampant inflammation of the pancreatic and peri-pancreatic tissues.^[18,19]^ Characterized by sudden onset, rapid progression, and elevated mortality rates, SAP is a significant threat to patient survival. The hypercatabolic stress state induced by the disease exacerbates protein and fat catabolism, compounded further by necessary periods of fasting, leading to malnutrition, immunosuppression, and increased susceptibility to infections.

A critical complication arising in the early stages of SAP is systemic inflammatory response syndrome, which exacerbates atrophy and necrosis of the intestinal mucosal cells, disrupts gut permeability, and consequently impairs the intestinal microbial barrier. Therefore, early enteral nutrition has been widely recognized for its effectiveness in SAP clinical management.^[20,21]^ The advantages of enteral nutrition stem from its ability to directly supply nutrients to the gut, thereby improving the patient’s nutritional state and supporting the integrity of the mucosal barrier to mitigate microbial translocation. However, while early enteral nutrition bolsters the gut barrier, it does not fully eradicate pathogenic bacteria. Probiotics, as live microbial formulations, are hypothesized to regulate the gut microbiota, suppress pathogenic bacterial proliferation, and reinforce the gut barrier.^[22,23]^ One study indicated that the concomitant application of probiotics with early enteral nutrition can not only restore gut functionality but also curb microbial translocation, thus alleviating SAP-induced inflammation.^[16]^

Our meta-analysis investigated the impact of probiotics on mortality, organ failure, and the duration of hospital stay in patients with SAP. Although we did not find a statistically significant reduction in mortality or risk of organ failure, an observed trend toward decreased mortality in the intervention group prompted further exploration. In particular, variations in the study methodology, patient demographics, and probiotic formulations may warrant future research to reconcile the observed heterogeneity. Significantly, we identified a robust and statistically meaningful reduction in the length of hospital stay of patients treated with probiotics, highlighting a potential avenue for enhancing patient care and optimizing healthcare resource allocation. This finding is supported by the low to moderate level of study heterogeneity, which suggests reliability. The rigor of these conclusions was further supported by a comprehensive sensitivity analysis, affirming the stability of the pooled results. While our findings delineate the complex potential of probiotics in the treatment of SAP, they underscore the need for additional well-designed studies to validate these initial observations and provide a comprehensive understanding of the clinical utility of probiotics in SAP management.

This meta-analysis had several limitations that should be acknowledged for a balanced interpretation of the findings. First, the moderate level of heterogeneity in mortality rates across the included studies suggests potential variability in the data. While specific sources of this heterogeneity were not pinpointed through subgroup analyses in our study, potential influences could arise from variations in study methodology, patient demographics, or the types of probiotic formulations used. Further investigations with subgroup or meta-regression analyses could help clarify these aspects. Second, the pooled sample size may not have been large enough to detect subtle but clinically significant benefits in outcomes, such as mortality or organ failure, thus potentially underestimating the impact of probiotics. Third, our meta-analysis did not assess the long-term outcomes and side effects of probiotic use, leaving an unaddressed gap that should be explored in future research to provide a comprehensive understanding of its clinical implications in the treatment of SAP.

## 5. Conclusions

In summary, this meta-analysis revealed that probiotics did not significantly reduce mortality or the risk of organ failure in patients with SAP. However, a noteworthy effect was observed in reducing the duration of hospital stay. These findings call for further studies are needed to explore the nuanced effects and potential long-term outcomes of probiotic therapy in this patient population.

## Author contributions

**Data curation:** Kui Jin.

**Formal analysis:** Zhiling Gao, Kui Jin.

**Methodology:** Shuomiao Yin, Longmei Wang.

**Investigation:** Shuomiao Yin.

**Resources:** Weiqun Nie, Ling Cheng.

**Supervision:** Ling Cheng.

**Validation:** Longmei Wang.

**Visualization:** Weiqun Nie.

**Writing – original draft:** Zhiling Gao.

**Writing – review & editing:** Zhiling Gao.
